# A Cyanotic Dilemma: Nitrobenzene Poisoning—A Case Report

**DOI:** 10.1002/ccr3.71329

**Published:** 2025-10-30

**Authors:** Janmejay Kumar Singh, Adrian Villaroman, Jaehoon Kim, Ghazal Majidi, Diana Stefanie Rojas Torres, Mrinal Bhandari, Fazeela Bibi, Khalil E. L. Abdi, Ujjwal Dutta, Sojitra Vani

**Affiliations:** ^1^ Teerthanker Mahaveer University Medical College and Research Centre Moradabad Uttar Pradesh India; ^2^ University of the East Ramon Magsaysay Memorial Medical Center Quezon City Philippines; ^3^ Catholic University of Daegu School of Medicine Daegu South Korea; ^4^ Tabriz University of Medical Sciences Faculty of Medicine Tabriz Iran; ^5^ The Lundquist Institute for Biomedical Innovation, Torrance Torrance California USA; ^6^ The Lundquist Institute for Biomedical Innovation Torrance California USA; ^7^ Jinnah Sindh Medical University Karachi Pakistan; ^8^ Faculty of Medicine and Pharmacy of Rabat Mohammed V University Rabat Morocco; ^9^ GMERS Medical College Gotri Vadodara India; ^10^ Bavadia Hospital Una India

**Keywords:** cyanosis, methemoglobinemia, methylene blue therapy, nitrobenzene poisoning

## Abstract

Acquired methemoglobinemia can rarely be caused by acute nitrobenzene poisoning, presenting as oxygen‐unresponsive, life‐threatening hypoxia. We discuss a case of a 19‐year‐old female who attempted suicide through intentional nitrobenzene poisoning. She presented to the emergency department with progressive cyanosis, severe abdominal pain, and dyspnea. Her symptoms were suggestive of methemoglobinemia, which was confirmed through arterial blood gas (ABG) analysis, revealing a methemoglobin level of 37.5%. Treatment included intravenous methylene blue, vitamin C, and supportive care, leading to clinical improvement. This case highlights the critical need for early recognition and timely administration of methylene blue, particularly in resource‐limited settings where delayed diagnosis can increase morbidity. It also signifies the importance of integrating psychiatric evaluation and mental health support in cases of intentional poisoning to prevent recurrence.

**Summary**

- Severe methemoglobinemia due to nitrobenzene poisoning can lead to life‐threatening hypoxia that is unresponsive to supplemental oxygen.
- Early recognition of characteristic signs, such as chocolate‐brown blood, is crucial for prompt diagnosis and timely treatment.
- Psychiatric evaluation is necessary in cases of self‐ingestion to prevent recurrence.

## 1 Introduction

Methemoglobinemia, a potentially life‐threatening condition, occurs when hemoglobin iron oxidizes to its ferric state, impairing oxygen delivery to tissues. Nitrobenzene, used in dyes, solvents, and industrial products, is a recognized cause of acquired methemoglobinemia through ingestion, inhalation, or dermal exposure [1]. Delayed diagnosis and treatment often result in high morbidity and mortality, particularly in resource‐limited settings [2].

Clinical manifestations range from mild cyanosis to severe hypoxia, metabolic acidosis, and multiorgan dysfunction. Chocolate‐brown arterial blood and elevated methemoglobin levels on arterial blood gas analysis (ABGA) are characteristic findings, with cyanosis unresponsive to supplemental oxygen [3]. Prompt treatment with methylene blue, a first‐line antidote, is critical, while adjunctive therapies such as high‐dose vitamin C, hydration, and symptom‐targeted management support recovery [4].

This report discusses a 19‐year‐old female who developed severe methemoglobinemia following intentional nitrobenzene poisoning. Delayed diagnosis and suboptimal initial treatment highlight the importance of early recognition, the challenges of nitrobenzene toxicity, and integrating psychiatric care into managing self‐harm cases.

## 2 Case History and Examination

A 19‐year‐old female with no previously known comorbidities presented to the emergency department with complaints of shortness of breath for 3 days (aggravated 4 h prior), bluish discoloration of fingers and face for 2 days, and severe abdominal pain for 1 day (Figure S1). She was admitted to a local hospital one week earlier after ingesting an unidentified poisonous substance following an argument with her husband. As stated by the attendants, after a few sessions of gastric lavage, she reported significant improvement in her symptoms and was therefore discharged the next day. Further inquiry revealed that the toxin consumed was a nitrobenzene compound.

The patient presented to the emergency department gasping for air, with severe abdominal pain accompanied by nausea, vomiting, and dizziness. She was conscious, with a Glasgow Coma Scale score of 13/15. She had labored respiration at 32 breaths per minute, a blood pressure of 124/70 mmHg, a pulse rate of 120 beats per minute, and an SpO_2_ of 50% on room air. Her blood sugar level was 172 mg/dL.

## 3 Diagnosis and Investigations

The clinical presentation raised suspicion of acute poisoning. Urgent abdominal ultrasonography revealed mild to moderate hepatic inflammation and ruled out any abdominal catastrophe. On ABGA, the blood sample appeared chocolate brown. The ABG report (Table 1) indicated a methemoglobin level of 37.5% and compensated metabolic acidosis.

**TABLE 1:** Arterial blood gas panel.

ABG analysis	Reference range	Day 1	Day 2	Day 3
pH	7.35–7.45	**7.32** (low‐normal)	**7.34** (low‐normal)	7.37
pCO_2_ (mmHg)	35–45	28 (compensated)	30	35
pO_2_ (mmHg)	80–100	**65**	**70**	**75**
HCO_3_^−^ (mEq/L)	22–28	**18**	**20**	**22**
Methemoglobin (%)	< 1.5	**35.5**	**25**	**10**

*Note:* The values in bold are the values outside of reference range.

There was no improvement with 100% oxygenation. Further investigations revealed normal chest and abdominal X‐rays, a normal ECG, normal serum creatinine and urea levels, and markedly elevated hepatic enzyme levels. A clinical diagnosis of acute severe methemoglobinemia due to nitrobenzene poisoning was made (Table 2).

**TABLE 2:** Serial laboratory data showing methemoglobinemia with transaminitis and anion gap metabolic acidosis.

Parameter	Reference range	Day 1	Day 2	Day 3
CBC				
Hemoglobin (g/dL)	12–15.5	13.2	13.1	13.1
Hematocrit (%)	35–47	41	40	40
WBC count (×10^3^/μL)	4–10	7.5	7.2	7.0
Platelets (×10^3^/μL)	150–400	260	250	245
Methemoglobin (%)	< 1.5	**37.5**	**25**	**11**
LFT				
AST (U/L)	10–40	**85**	**75**	**65**
ALT (U/L)	7–56	**75**	**67**	**61**
Total bilirubin (mg/dL)	0.1–1.2	**1.7**	**1.5**	1.3
Direct bilirubin (mg/dL)	0–0.3	**0.6**	**0.5**	0.4
ALP (U/L)	44–147	100	95	90
Albumin (g/dL)	3.5–5.5	3.8	3.7	3.7
CMP/BMP				
Sodium (mEq/L)	135–145	138	137	137
Potassium (mEq/L)	3.5–5.0	4.6	4.5	4.4
Chloride (mEq/L)	98–107	103	102	101
Bicarbonate (mEq/L)	22–28	**18**	**20**	**22**
BUN (mg/dL)	7–20	12	13	13
Creatinine (mg/dL)	0.6–1.2	0.8	0.9	0.9
Glucose (mg/dL)	70–99 (fasting)	95	92	90
Calcium (mg/dL)	8.6–10.3	9.2	9.1	9.0
Anion gap (mEq/L)	8–12	**16**	**14**	12

*Note:* The values in bold are the values outside of reference range.

## 4 Treatment and Follow‐Up

The patient was treated with intravenous methylene blue (100 mL of a 1% sterile solution), administered twice daily at 12‐h intervals. The treatment significantly improved her SpO_2_ to 92%. Intravenous vitamin C, vitamin K, 10% dextrose, and IV antibiotics were also added. Urine output was maintained at 100 mL/h alongside normal central venous pressure through intravenous hydration.

With treatment, the patient's condition stabilized, and her SpO_2_ improved to 96% by day 5. The patient was discharged on day 7 with iron, folate, and vitamin C supplements. Psychiatric evaluation led to a diagnosis of depression, and she was started on antidepressants. She was scheduled for follow‐up in two weeks for both medical and psychiatric care.

## 5 Discussion

### 5.1 Significance of the Case

This case highlights the diagnostic and therapeutic challenges of severe methemoglobinemia caused by nitrobenzene poisoning, particularly in a resource‐limited setting. Methemoglobinemia is a rare but life‐threatening condition that requires early recognition and prompt intervention to avoid significant morbidity or mortality. In this case, the delayed diagnosis was compounded by inadequate documentation and an incomplete initial workup, which reflects common barriers in rural healthcare facilities. Despite these challenges, the clinical clues—such as cyanosis unresponsive to supplemental oxygen, ABG findings of low oxygen saturation despite a normal PaO_2_, and the chocolate‐brown blood appearance—were pivotal in leading to the correct diagnosis and subsequent treatment.

Similar findings have been reported in prior cases of nitrobenzene poisoning, where timely detection of chocolate‐brown discoloration of blood facilitated accurate diagnosis [5].

The successful outcome following methylene blue administration and supportive care underscores the importance of timely and appropriate management. Furthermore, the case brings attention to the often‐overlooked psychosocial factors, including the patient's psychiatric history and the circumstances surrounding the poisoning. This multifaceted case reinforces the need for a holistic approach that integrates acute care, toxicological expertise, and mental health support, particularly in vulnerable populations.

Numerous case reports have reported nitrobenzene‐induced methemoglobinemia, which frequently presents with diagnostic difficulties similar to this one. In a study by Smith et al. [6] For example, methemoglobinemia was first overlooked because co‐oximetry was not available for a patient with severe cyanosis and chocolate‐brown blood. The diagnosis was only confirmed in our case after persistent hypoxemia despite normal PaO_2_, highlighting the need for increased clinical suspicion when ABG and pulse oximetry results are inconsistent. A fatal outcome in a patient who received delayed methylene blue administration was highlighted in another report by Lee et al. [7], highlighting the importance of prompt intervention for survival. Our patient's successful outcome was mirrored in a case from a tertiary care center [8], which showed quick recovery as a result of early detection and treatment. Together, these cases highlight the fact that, despite consistent clinical presentation, timely diagnosis, and the availability of resources are critical to successful outcomes. As demonstrated here, rural facilities might only use clinical hints, in contrast to urban centers with toxicology expertise. Furthermore, the psychiatric aspect of purposeful nitrobenzene ingestion, which our patient experienced, has been documented in other instances [9], indicating the necessity of integrated mental health evaluations in poisoning treatment.

### 5.2 Pathophysiology

Nitrobenzene causes oxidation of hemoglobin iron from the ferrous (Fe^2+^) state to the ferric (Fe^3+^) state, inducing oxidative stress and forming methemoglobin, which cannot bind oxygen. This leads to “functional anemia,” where oxygen delivery to tissues is severely impaired, resulting in hypoxia and acidosis despite adequate oxygenation.

Secondary pathophysiology includes the transformation of nitrobenzene to intermediate byproducts that cause red blood cell rupture and hemolysis. The byproducts decrease the amount of reduced glutathione. Blood cell survival depends crucially on reduced glutathione, as it maintains the normal function of the red blood cell membrane. Therefore, its depletion leads to hemolysis. The basophilia in globin molecules is also impacted directly by the intermediate products, leading to their denaturation. Inclusion bodies (Heinz corpuscles) in red blood cells are also seen because of condensed, precipitated, denatured globin. These red blood cells are prone to rupture, further contributing to hemolysis [10].

In the case described above, the ingestion of nitrobenzene led to significant methemoglobinemia, confirmed at 37.5% on ABG, accounting for the cyanosis and tissue hypoxia noted despite supplemental oxygen therapy. Early detection is crucial for proper management.

Nitrobenzene induces oxidative stress and forms methemoglobin, which is unable to bind oxygen, by oxidizing hemoglobin iron from the ferrous (Fe^2+^) state to the ferric (Fe^3+^) state. This results in “functional anemia,” a condition in which tissues' ability to receive oxygen is significantly compromised, leading to hypoxia and acidosis even when oxygenation is adequate. Comparable processes have been documented in dapsone and aniline poisoning cases, where coma and cardiovascular collapse were linked to methemoglobinemia levels above 30% [11]. The quick onset of severe symptoms seen in this instance can be explained by the fact that nitrobenzene has a higher oxidative potency than other aromatic nitro compounds [12].

### 5.3 Diagnosis

Limited resources and subpar toxicology testing in a rural hospital setting led to a delay in diagnosis. Initial treatment with gastric lavage did not address the underlying methemoglobinemia. Limited knowledge about rare poisoning cases, such as nitrobenzene compounds, was also a reason for delayed recognition.

The patient presented with cyanosis that did not respond to 100% oxygen therapy, a pathognomonic feature of methemoglobinemia. The arterial blood's appearance of chocolate‐brown color on ABG analysis provided a strong visual clue. ABG findings confirmed the diagnosis through elevated methemoglobin levels of 37.5%, highlighting the importance of specific testing in suspected toxic exposures. Literature emphasizes these clinical findings as key factors for the early detection of the condition.

### 5.4 Treatment

Methylene blue, being an electron acceptor and reducing agent, catalyzes the reduction of methemoglobin back to hemoglobin through the NADPH‐dependent pathway [10]. In this particular case, the dosing intervals of 12 h were clinically appropriate and aligned with the level of severity (37.5% methemoglobin). The success of therapy was signified by the increase in SpO_2_ levels from 50% to 92% following the initial dose, with levels returning to normal at 96% by day 5.

Blue‐green urine was observed within 3–6 h of initiating treatment with methylene blue [13]. The color returned to normal in approximately 72–96 h. The metabolism of methylene blue into leucomethylene blue by nicotinamide adenine dinucleotide (NADH), which is excreted in urine, imparted the urine's blue‐green color [14]. Treatment in this case aligns with standard protocols, as methylene blue is the first‐line therapy for methemoglobin levels higher than 30%.

Vitamin C (ascorbic acid) played its part as a potent antioxidant, reducing oxidative stress [15]. Supportive treatment with hydration, IV antibiotics, and folate/iron contributed to the patient's rapid recovery.

Hyperbaric oxygen or exchange transfusion was not required, as this patient's initial methemoglobin level was below 50%, and she responded well to basic treatment [16, 17].

Through the NADPH‐dependent methemoglobin reductase pathway, methylene blue, an electron acceptor and reducing agent, catalyzes the conversion of methemoglobin (MetHb) to hemoglobin [18]. In extreme situations like this one, where the initial MetHb level was 37.5%—much higher than the 30% threshold requiring treatment [19]—this mechanism is especially important. The 12‐h dosing interval was clinically appropriate and consistent with Gupta et al.'s recommendations for MetHb > 30%, where repeated doses are frequently required because of the prolonged oxidative effects of nitrobenzene [20]. According to studies demonstrating response times of 30 to 60 min in methylene blue‐sensitive cases, the therapeutic success was demonstrated by the quick improvement in SpO_2_ from 50% to 92% after the first dose [21]. The drug's effectiveness when taken early was demonstrated by the fact that SpO_2_ normalized to 96% by day 5.

A well‐known adverse effect of renal excretion of leucomethylene blue (a reduced metabolite via NADH) is blue‐green urine, which is seen 3–6 h after methylene blue initiation [22]. According to a case series by Ng et al., this discoloration usually goes away in 72–96 h [23]. Appropriate dosage was further validated by the lack of paradoxical methemoglobinemia, a rare side effect of high‐dose methylene blue [24]. Adjuvant treatments were crucial, like ascorbic acid or vitamin C, which showed to be a strong antioxidant in cases of refractory methemoglobinemia, reducing oxidative stress and promoting glutathione regeneration [25]. In accordance with management guidelines for toxin‐induced hemolytic anemia, supportive care (IV hydration, folate/iron, and antibiotics) addressed secondary hemolysis and avoided infections [26].

### 5.5 Hepatic Involvement and Organ‐Specific Toxicity

Nitrobenzene poisoning primarily induces methemoglobinemia but can also cause hepatotoxicity, as evidenced by elevated hepatic transaminases. This suggests hepatocellular injury consistent with oxidative stress on hepatocytes, a mechanism described by Cichoż‐Lach and Michalak [27]. Nitrobenzene is metabolized in the liver to toxic intermediates, generating reactive oxygen species (ROS) that damage cells [28].

Hepatotoxicity, often presenting as transient liver enzyme elevation, has been well documented in poisoning cases [29]. Severe methemoglobinemia‐induced hypoxia may exacerbate liver injury through ischemic insult. Excluding other causes such as ischemic hepatitis, sepsis, or drug‐induced liver injury strengthens the association with nitrobenzene exposure.

Although liver function abnormalities were self‐limiting in this case, they highlight the need for vigilant monitoring of multiorgan involvement. Supportive measures like hydration can help mitigate further organ damage. This case underscores the systemic effects of nitrobenzene and the importance of comprehensive clinical monitoring beyond methemoglobinemia resolution.

### 5.6 Psychiatric and Social Dimensions

There are three studies based on the psychiatric and social factors that we should focus on to deal with deliberate self‐poisoning.

The study on deliberate self‐poisoning in Bangladesh shows that the vulnerable age, mostly under age 30, particularly teenagers, is more often the victims of deliberate self‐poisoning with multiple issues like financial, academic, unemployment, and domestic problems.

It also shows that other factors like gender ratio are skewed more toward women, with a 1:1.35 gender ratio, indicating that females are more affected by psychiatric suffering as they are vulnerable to domestic violence [30].

The economic gap with deliberate self‐poisoning (Paraquat) has been shown by one study in northeast Colombia, representing regions with unequal land distribution as a profound factor. Limited accessibility to resources for poor farmers, coupled with inadequate knowledge of the safe use of pesticides and insufficient health insurance, increases their vulnerability [31].

As our case report shows, the 19‐year‐old female with a history of arguments with her husband, the social factors above should be accounted for in patient care.

Deliberate self‐poisoning accounts for an important social factor as well. Psychiatric disorders are mainly the issue, with schizophrenia and depression accounting for 30% of cases in the deliberate self‐poisoning study in Bangladesh. On top of that, the type of agents used depends on the setting, as rural regions show 59% of cases involving insecticides, whereas prescription drugs dominate in urban areas. However, in terms of deliberate self‐poisoning, sedatives are the most common agents used, with pesticides being the second most used [30].

With these factors, one prospective study, involving a clinical trial of 312 cases from 276 patients with self‐poisoning, highlights what psychiatrists and medical teams can do to provide better care services during and after hospital admission. This includes dedicated training for the medical team to tackle multiple problems, such as social, physical, and psychiatric issues [32].

Out of this result, the findings emphasize the need for enhanced mental health programs with education, stricter control of poisoning agents, community‐based solutions, and government interventions to deal with deliberate self‐poisoning risks and improve outcomes. The case we report does not consider the social complexity of the history, diagnosis, and treatment of deliberate self‐poisoning, which led to delays in treatment and a lack of attention to psychiatric and social dimensions. This limitation was obvious.

After all, the three studies above, along with their statistics, make it evident that governments, physicians, and members of society must thoroughly address the prevention and treatment of deliberate self‐poisoning cases through multiple social measures that should not be overlooked.

### 5.7 Benchmarking the Case With Existing Literature

There are two cases that we can focus on regarding the timing of treatment for methemoglobinemia in comparison with the case report, which indicates the importance of prompt treatment. A case with a 21‐year‐old male patient who consumed pesticide with emamectin benzoate and indoxacarb showed dramatic results on methemoglobinemia after receiving methylene blue injection, intravenous Vitamin C (2.0 g), and hemoperfusion preparation [33]. This was similar to a case with a 20‐year‐old female patient at a rave party showing amphetamine and cocaine positivity on a drug screen, with drastic improvement within 8 h after receiving a 1 mg/kg methylene blue infusion [34].

Each case showed methemoglobinemia with methemoglobin levels of 32.9% and 30%, respectively, confirmed by ABGA, which was crucial for diagnosis and preparation for treatment. In the female patient, methemoglobin levels normalized to 3.5%, while in the male patient, methemoglobin levels normalized within 24 h after arrival, with transfer out of the intensive care unit at 48 h.

Additionally, each case exhibited symptoms related to methemoglobinemia, including systemic symptoms like lethargy, dizziness, headache, respiratory issues such as shortness of breath, or GI symptoms like abdominal pain. While these symptoms can aid in diagnosis, they may also be ambiguous unless accompanied by specific signs, such as the brown arterial and venous blood observed in the 21‐year‐old male with pesticide ingestion.

Thus, focusing on each case's history is essential for guiding the next steps, such as ABGA, which can aid in diagnosis and prompt treatment.

In our case report, significant delays in treatment were observed due to nitrobenzene compound consumption. Only a couple of gastric lavage sessions were performed after immediate ingestion, underscoring the importance of history taking and promptness in treatment. Identifying the substance as a nitrobenzene compound earlier would have allowed for a rapid diagnosis and treatment, preventing symptom aggravation over several days, as compared with the two cases above.

## 6 Conclusion

A case of intentional nitrobenzene poisoning causing severe methemoglobinemia highlights the importance of early diagnosis and timely treatment to prevent morbidity and mortality. The delay in this patient's diagnosis and suboptimal treatment underscores the limitations of healthcare in resource‐limited settings. Symptoms of oxygen‐unresponsive cyanosis and the appearance of chocolate‐brown‐colored blood raise clinical suspicion of methemoglobinemia, which is further confirmed by ABGA. Immediate treatment with methylene blue is life‐saving for patients. Additionally, in cases of intentional poisoning, psychiatric evaluation and mental health support are crucial for preventing recurrence. Addressing both the medical and psychosocial aspects of such cases is vital for comprehensive patient care and long‐term well‐being.

In resource‐constrained settings, where methemoglobinemia is frequently misdiagnosed due to reliance on pulse oximetry alone, this case also highlights the urgent need for enhanced diagnostic protocols. Delays could be minimized by teaching physicians to identify classic clinical signs (such as chocolate‐brown blood and SpO_2_‐PaO_2_ discordance) and by promoting point‐of‐care co‐oximetry in high‐risk areas. To close care gaps, standardized treatment algorithms—such as weight‐based methylene blue dosage and repeat administration criteria—must be given top priority.

In order to address potential complications like residual hemolysis or the neurocognitive effects of prolonged hypoxia, long‐term follow‐up is crucial after acute management. A multidisciplinary strategy that incorporates social services, psychiatry, and toxicology can reduce the likelihood of recurrence in cases of intentional poisoning. The prevalence of such avoidable tragedies may be further decreased by public health initiatives that focus on mental health awareness and the regulation of toxic substances.

## Author Contributions

**Janmejay Kumar Singh:** conceptualization, supervision, writing – original draft. **Adrian Villaroman:** project administration, resources, writing – review and editing. **Jaehoon Kim:** writing – original draft, writing – review and editing. **Ghazal Majidi:** supervision, visualization, writing – review and editing. **Diana Stefanie Rojas Torres:** investigation, writing – review and editing. **Mrinal Bhandari:** investigation. **Fazeela Bibi:** writing – review and editing. **Khalil E. L. Abdi:** writing, final review and editing and proofreading. **Ujjwal Dutta:** investigation. **Sojitra Vani:** investigation.

## Ethics Statement

The authors have nothing to report.

## Consent

Written consent was taken from the patient. The patient is in contact with the authors.

## Conflicts of Interest

The authors declare no conflicts of interest.

## Supporting information


**Figure S1:** Examination: extremities as well as tongue was bluish in color.

## Data Availability

The data was taken from a patient who presented to our hospital; all data and references are publicly available on databases such as PubMed and Google Scholar.
