# Supplementary figures and images for: A Cyanotic Dilemma: Nitrobenzene Poisoning—A Case Report
Source: Clin Case Rep. 2025 Oct 30;13(11):e71329. doi: 10.1002/ccr3.71329 (PMC12575183; doi:10.1002/ccr3.71329)

**Examination: extremities as well as tongue was bluish in colour**


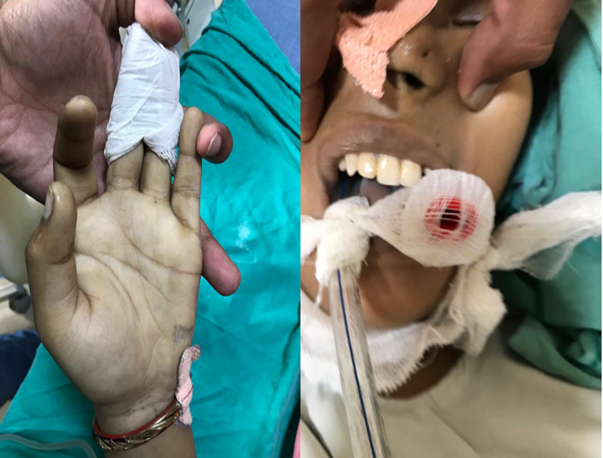

Supplement: Supplementary file 1 — Figure S1: Examination: extremities as well as tongue was bluish in color. [file CCR3-13-e71329-s001.docx]
